# Antioxidant and *α*-Glucosidase Inhibitory Activities Guided Isolation and Identification of Components from Mango Seed Kernel

**DOI:** 10.1155/2020/8858578

**Published:** 2020-12-28

**Authors:** Dan Yang, Xuee Chen, Xida Liu, Na Han, Zhihui Liu, Sikai Li, Jianxiu Zhai, Jun Yin

**Affiliations:** Department of Pharmacognosy and Utilization Key Laboratory of Northeast Plant Materials, School of Traditional Chinese Medicine, Shenyang Pharmaceutical University, Shenyang 110016, China

## Abstract

In the present study, petroleum ether, dichloromethane, ethyl acetate, and *n*-butanol fractions of mango seed kernel exhibited different degrees of antioxidant and *α*-glucosidase inhibitory activity. Thus, quantitative and qualitative analysis of the petroleum ether fraction was conducted by GC-MS. Among identified components, four unsaturated fatty acids had never been reported in natural products before, together with 19 known components. In addition, 17 compounds were isolated and elucidated from other active fractions. Compounds **2**, **9**, **15**, and **17** were isolated for the first time from *Mangifera* genus. Compounds **1** and **2** exhibited prominent DPPH radical scavenging and *α*-glucosidase inhibitory effects. In order to further explore their mechanism of *α*-glucosidase inhibition, their enzyme kinetics and in silico modeling experiments were performed. The results indicated that **1** inhibited *α*-glucosidase in a noncompetitive manner, whereas **2** acted in a competitive manner. In molecular docking, the stability of binding was enhanced by *π*-*π* T-shaped, *π*-alkyl, *π*-*π* stacked, hydrogen bond, and electrostatic interactions. Thus, compounds **1** and **2** were determined to be new potent antioxidant and *α*-glucosidase inhibitors for preventing food oxidation and enhancing hypoglycemic activity.

## 1. Introduction

Type 2 diabetes is a chronic and complex metabolic disease characterized mainly by hyperglycemia, insulin resistance, and insufficient insulin secretion [1]. When carbohydrates are swallowed, dietary polysaccharides are absorbed by the hydrolysis effects of enteral *α*-glucosidase to obtain free monosaccharides [2]. Free monosaccharides assimilated in the blood can lead to hyperglycemia caused by type 2 diabetes [3]. One of the diabetes treatments is to inhibit the enzyme activity of *α*-glucosidase to minimize the formation of blood glucose. *α*-Glucosidase inhibitors extend the overall carbohydrate digestion time and decrease the absorption of glucose, which slow postprandial glucose elevation [4]. Several drugs, *α*-glucosidase inhibitors, such as voglibose, acarbose, and miglitol, are now available to treat the patients who suffer from postprandial hyperglycemia. However, a long-term use of these inhibitors has been associated with severe side effects, such as diarrhea and vomiting [5]. Hyperglycemia-induced oxidative stress is implicated in the onset and progression of diabetes and, if left untreated, can lead to severe complications [6]. Free radicals and reactive oxygen species are products of normal cellular metabolism and are extremely reactive and potentially damaging transient chemical species [7]. Numerous studies showed that over generation of free radicals and reactive oxygen species could be harmful to human health and trigger many diseases, such as cancer, arteriosclerosis, inflammatory disorders, and aging processes [7]. Therefore, the development of high-efficiency and low-toxicity antioxidant and *α*-glucosidase inhibitors from natural resources has become one research hotspot.

Mango (*Mangifera indica* L.) is one of the most popular fruit worldwide, which belongs to the Anacardiaceae and grown in many places around the world, particularly in tropical and subtropical countries. This fruit is available as a dietary supplement due to their high nutritional value, such as providing dietary fiber, fats, proteins, carbohydrates, and phenolic compounds, which are vital to human growth, development, and health [8]. Mango seed kernel, a traditional Chinese medicine, is widely available and used in China, and its bioactivities had been recorded. Chaianun and Praphan [9] indicated that the 95% ethanol extracts from two different kinds of mango seed kernel, namely, *Kaew* and *Choke-Anan*, both had good *α*-glucosidase inhibitory activity with IC_50_ values of 163.19 ± 2.33 and 113.51 ± 5.85 *μ*g/mL, respectively. Emmanuel, Ganiyu, Afolabi, Aline, and Margareth [10] had demonstrated that the methanol extract of mango seed kernel could significantly inhibit the activity of *α*-glucosidase. Although it was used for centuries as a traditional folk prescription in China, there are few studies on chemical components in mango seed kernel and bioactivities of its monomeric compounds. Meanwhile, no investigations on the composition and activity of its petroleum ether fraction have been previously reported.

Consequently, a bioassay-guided fractionation method was used by us to research the bioactive components in mango seed kernel. Its crude extract was successively partitioned with petroleum ether, dichloromethane, ethyl acetate, and *n*-butanol, respectively. The ethyl acetate and *n*-butanol fractions showed significant DPPH radical scavenging activity. Petroleum ether, dichloromethane, and ethyl acetate fractions revealed moderate *α*-glucosidase inhibitory activity. These finding prompted us to perform a detailed chemical evaluation, leading to the identification of 23 components from the petroleum ether fraction by LC-MS and the isolation and purification of 17 compounds from other active fractions of mango seed kernel. Antioxidant and *α*-glucosidase inhibitory activities of the identified components from the petroleum ether fraction had been reported in many references. Bioactivities of 13 isolated compounds from other active fractions were tested, and compounds **1** and **2** were found to be active. The enzyme kinetic parameters of active compounds were also determined. The results of the molecular docking were used to visualize how they bind to *α*-glucosidase, providing insights into *α*-glucosidase inhibition mechanism of the active compounds.

## 2. Materials and Methods

### 2.1. Chemicals and Reagents

1D NMR spectra was measured on a bruker ARX-600 spectrometer using tetramethylsilane as an internal standard. Absorbance data were recorded on KHB-ST-360 ELIASA (Shanghai Kehua Bio-engineering Co., Ltd.). Preparative chromatography was performed on a CHEETAH Flash MP200, while semipreparative HPLC was performed on a Shimadzu LC-10A spectrophotometer, using a Welch XB-C18 semipreparative column (5 *μ*m, 10 × 250 mm). Column chromatographies (CC) were carried out over silica gel (200–300 mesh, Qingdao Haiyang Chemical Co., Shandong, China), ODS (50–100 mesh, YMC, Co., Ltd., Japan), and Sephadex LH-20 (Pharmacia, USA). Gas chromatography mass spectrometry (GC-MS) spectra were recorded on a Shimadzu (TQ-8040) series GC-MS system (n). *α*-Glucosidase from Baker's yeast was purchased from Shanghai Yuanye Biotechnology Co., Ltd. Acarbose was purchased from Hangzhou Zhongmei Huadong Pharmaceutical Co., Ltd. *p*-NPG (*p*-nitrophenyl-*α*-D-glucopyranoside) was purchased from Shanghai McLean Biochemical Technology Co., Ltd. DPPH (1,1-diphenyl-2-picrylhydrazyl) and anhydrous Na_2_CO_3_ were obtained from Sigma Aldrich (St. Louis, MO, USA). Deuterium generation reagents were obtained from Cambridge Isotope Laboratories, Inc. Dipotassium phosphate was obtained from Junsei Chemical Co., Ltd. (Tokyo, Japan). Monopotassium phosphate was purchased from Yakuri Pure Chemicals Co., Ltd. (Osaka, Japan), and all other chemicals and solvents of analytically pure and HPLC grade were purchased from Shenyang Lebo Reagent Co. Ltd (Shenyang, China).

### 2.2. Plant Material

The seeds of *Mangifera indica* L. were obtained from Shenyang Guoda Pharmacy Co., Ltd. (Liaoning Province, China, in 2017). The botanical identification was made by Prof. Jun Yin of Shenyang Pharmaceutical University. A voucher specimen (No. 20170920) was deposited in the Research Department of Traditional Chinese Medicine, Shenyang Pharmaceutical University, Shenyang, Liaoning, China.

### 2.3. Antioxidant and *α*-Glucosidase Inhibitory Activities Evaluation of the Extract and Fractions from Mango Seed Kernel

The seeds of *Mangifera indica* L. (1 kg) were removed from the shell, which homogenized with a homogenizer. Dried powders (600 g) of mango seed kernel were exhaustively extracted with 60% ethanol for 1 h (20 L × 2 times) at room temperature and were concentrated under reduced pressure. The concentrated solution was successively partitioned with petroleum ether, dichloromethane, ethyl acetate, and *n*-butanol.

#### 2.3.1. DPPH Assay

The DPPH radical scavenging activity assay was determined according to the reported method with minor modifications [11]. Briefly, extract and four fractions were dissolved in methyl alcohol at concentrations ranging from 62.5 to 1000 *μ*g/mL, which were prepared to mix with a methyl alcohol solution of DPPH (0.2 mM, 100 *μ*L) on a 96-well plate. After that, shaken gently and stood at room temperature for 30 min, the optical density (OD) 517 nm in each well were determined by a microplate reader. Triplicates of each sample were run, and the mean values were calculated. Ascorbic acid was chosen as positive control. The percentage inhibition (%) for each sample was calculated by the following formula:

(1) $$\begin{array}{c} \text{Inhibition }\left(\%\right)=\left[\frac{1-\left({\text{Abs}}_{\text{samples}}-\text{Ab}{\text{s}}_{\text{control}}\right)}{{\text{Abs}}_{\text{blank}}}\;\right]\times 100\%. \end{array}$$

DPPH solution was replaced by a methanol solution, which was used as control, and samples were replaced by the methanol solution as the black. All the results were expressed as means ± standard deviation (SD). IC_50_ values denoted the concentration of the sample required to scavenge 50% of the DPPH radical.

#### 2.3.2. *α*-Glucosidase Inhibitory Assay

The *α*-glucosidase inhibitory assay was performed on a 96-well plate by using a microplate reader according to the published method [12] with minor modifications. Extract and four fractions were required to dissolve in 5% DMSO at the concentrations ranging from 62.5 to 1000 *μ*g/mL. In brief, 20 *μ*L of *α*-glucosidase (1.3 U/mL) and 20 *μ*L sample were added to 20 *μ*L of 0.1 M potassium phosphate buffer (pH 6.8). After 5 min incubation at 37°C, 20 *μ*L of 4-*p*NPG (*p*-nitrophenyl-*α*-D-glucopyranoside) (2.5 mM) was added and incubated for 15 min; then, 80 *μ*L of Na_2_CO_3_ (0.2 M) was added to stop the reaction. The optical density (OD) values were determined using a microplate reader at 405 nm. Triplicates of each sample were run, and the mean values were calculated. Acarbose was used as positive control. The percentage inhibition (%) was calculated as the following formula:

(2) $$\begin{array}{c} \text{Inhibition }\left(\%\right)=\left[\frac{1-\left(A_{\text{a}}-A_{\text{b}}\right)}{A_{\text{c}}-A_{\text{d}}}\right]\times 100\%.\\ A_{\text{a}}:\text{absorbance of the sample group with enzyme}.\\ A_{\text{b}}:\text{absorbance of the sample control group without enzyme}.\\ A_{\text{c}}:\text{absorbance of the control group without samples}.\\ A_{\text{d}}:\text{absorbance of the blank control group without samples and enzyme}. \end{array}$$

The IC_50_ was calculated by using SPSS software.

### 2.4. GC-MS Analysis of the Bioactive Fraction (Petroleum Ether Fraction) from Mango Seed Kernel

The GC-MS analysis of constitutes in the bioactive fraction (Petroleum ether fraction) of mango seed kernel was carried out on a Shimadzu (TQ-8040) series GC-MS system (n) equipped with an AOC-20i autosampler [13]. The operational process of GC-MS analysis was as follows: rising from 40°C to 130°C at a rate of 4°C/min and holding for 2 min, rising from 130°C to 150°C at a rate of 2°C/min and holding for 3 min, rising from 150°C to 180°C at a rate of 2°C/min and holding for 3 min, and finally enhanced to 210°C and held isothermally for 5 min. Chemical constituents were then pointed out based on the mass spectra and retention times with already known compounds in the NIST14 and NIST14s (National Institute of Standards and Technologies, Mass Spectra Libraries, Gaithersburg, MD, USA) [13].

### 2.5. Isolation and Purification of Compounds in Active Fractions from Mango Seed Kernel

The dichloromethane fraction (4.2 g) was subjected to a silica gel column (200-300 mesh) in gradient elution of mixture solvent composed of petroleum ether-acetone (from 100 : 10 to 100 : 100, *v*/*v*) and led to four subfractions (Fr.1-1-Fr.1-4). Compounds **10** (22.2 mg) and **7** (2.4 mg) were yielded through recrystallization (MeOH) from Fr.1-2, and the remaining fraction was further purified by semipreparative HPLC (MeOH-H_2_O, 45 : 55) to obtain compound **13** (2.1 mg). Fr.1.3 was further separated repeatedly with Sephadex LH-20 (MeOH) to obtain two subfractions (Fr.1.3.1- Fr.1.3.2). Compound **6** (100.1 mg) was obtained through recrystallization (MeOH) from Fr.1.3.1. Fr.1.3.2 was subjected to preparative TLC (CH_2_Cl_2_-Acetone, 10 : 8) to obtain compound **8** (2.0 mg). Fr.1.4 was further separated repeatedly with Sephadex LH-20 (MeOH) to obtain compound **16** (2.3 mg).

The ethyl acetate fraction (19.6 g) was subjected to a silica gel column chromatography (200-300 mesh) and was eluted in a gradient manner with CH_2_Cl_2_/MeOH (from 50 : 1 to 3 : 1, *v*/*v*) to yield three subfractions (Fr.1-Fr.3). Fr.2 was separated by Sephadex LH-20 (MeOH) to obtain two subfractions (Fr.2-1-Fr.2-2). Fr.2-1 was isolated by preparative TLC (EtOA_C_-MeOH-H_2_O, 5 : 1 : 0.9) to obtain compound **14** (2.1 mg). Fr.2-2 was further purified by semipreparative HPLC (MeOH-H_2_O, 35 : 65) to obtain **15** (2.0 mg). Fr.3 was partly separated by Sephadex LH-20 (MeOH) to obtain some subfractions, compounds **1** (420.0 mg) and **5** (10.2 mg). One of the subfractions was subjected to preparative TLC (EtOA_C_-MeOH-H_2_O, 5 : 1 : 0.9) to obtain compounds **11** (2.1 mg) and **12** (2.2 mg). Another part of Fr.3 was chromatographed on a reversed-phase ODS by gradient elution with MeOH/H_2_O (10 ⟶ 70%, *v*/*v*) to obtain five subfractions (Fr.3.1-Fr.3.5). Fr.3.3 was separated repeatedly with Sephadex LH-20 (MeOH) to obtain two subfractions (Fr.3.3.1-Fr.3.3.2). Fr.3.3.1 was further purified through preparative TLC (EtOAc-MeOH-H_2_O, 5 : 1 : 0.9) to obtain compound **4** (8.3 mg). Fr.3.3.2 was chromatographed over semipreparative HPLC (MeOH-H_2_O, 35 : 65) to obtain compound **3** (2.3 mg). Fr.3.4 was separated by semipreparative HPLC (MeOH-H_2_O, 40 : 60) to obtain compound **2** (4.4 mg).

The *n*-butanol fraction (25.3 g) was subjected to a reversed-phase ODS, and elution with a gradient of MeOH (5 ⟶ 50%, *v*/*v*) in water to obtain four subfractions (Fr.1-Fr.4). Compounds **9** (6.2 mg) and **17** (5.2 mg) were obtained from Fr.1 by preparative TLC (EtOAc-MeOH-H_2_O, 5 : 1 : 0.9).

1, 2, 3, 4, 6-Penta-*O*-galloyl-*β*-D-glucoside (**1**): maple powder; ^1^H and ^13^C NMR data matched literature values [14].

1, 2, 3, 4, 6-Penta-*O*-galloyl-*α*-D-glucoside (**2**): maple powder; ^1^H and ^13^C NMR data were in agreement well with published data [15].

1, 2, 3, 6-Tetra-*O*-galloyl-*β*-D-glucoside (**3**): white powder; ^1^H and ^13^C NMR data were similar to the literature values of 1, 2, 3, 6-Tetra-*O*-galloyl-*β*-D-glucoside [16].

1-*O*-(3, 4, 5-Trihydroxybenzoyl)-*β*-D-glucoside (**4**): white powder; ^1^H and ^13^C NMR data were in agreement with the literature values of 1-*O*-(3, 4, 5-Trihydroxybenzoyl)-*β*-D-glucose [17].

Methyl galate (**5**): yellow Amorphous powder; ^1^H and ^13^C NMR data agreed well with literature values [18].

Gallic acid (**6**): white needle crystal; ^1^H and ^13^C NMR data matched literature values (Ni, Liang, Huang, Chen, Zou, Li, et al., 2019).

3,4-Dihydroxybenzoic acid (**7**): colorless needle crystal; ^1^H and ^13^C NMR data were identical to those recorded by Chunhakant and Chaicharoenpong [19].

4-Hydroxybenzoic acid (**8**): colorless needle crystal; ^1^H and ^13^C NMR data were identical to those reported [20].

1-Glycerol gallate (**9**): pale yellow powder; ^1^H and ^13^C NMR data were in agreement with the literature values [21].

Ethyl gallate (**10**): colorless powder; ^1^H and ^13^C NMR data were similar to those recorded by Chen et al. [22].

Ferulic acid (**11**): white powder; ^1^H and ^13^C NMR data matched literature values [23].

Caffeic acid (**12**): white needle crystal; ^1^H and ^13^C NMR data were in agreement with those dealt with in the literature [24].

Quercetin (**13**): yellow powder; ^1^H and ^13^C NMR data were identical to those reported [25].

Genistein (**14**): pale yellow powder; ^1^H and ^13^C NMR data matched literature values [26].

Gallocatechin (**15**): yellow amorphous powder; ^1^H and ^13^C NMR data were identical to those recorded by Foo, Lu, Molan, Woodfield, and McNabb [27].

Epicatechin (**16**): white powder; ^1^H and ^13^C NMR data were in agreement well with published data (M. [28]).

Hexyl-*β*-D-glucoside (**17**): pale yellow powder; ^1^H and ^13^C NMR data agreed well with literature values [29].

### 2.6. Determination of Antioxidant and *α*-Glucosidase Inhibitory Activities of the Isolated Compounds

The antioxidant and *α*-glucosidase inhibitory activities of each isolated compound at concentrations ranging from 15.625 to 500 *μ*M (DPPH) and from 125 to 2000 *μ*M (*α*-glucosidase inhibitory), respectively, were determined using the methods described in Section 2.3.

### 2.7. Inhibitory Kinetic Analysis

The kinetic mechanisms of active compounds **1** and **2** towards *α*-glucosidase were determined by the graphical views of *Dixon and Lineweaver*-*Burk plots* [30]. The concentration ranges for the substrates were 0.8-5.0 mM. The concentration of the enzyme was kept constant at 1.3 U/mL. Lineweaver-Burk plots were established to evaluate the type of inhibition. Dixon plots were used to calculate inhibitory constant (*K*_i_) values of the tested compounds. Each kinetic analysis was implemented in triplicate from which mean *K*_i_ values.

### 2.8. Molecular Docking Calculation

To investigate the interaction between active compounds and *α*-glucosidase, molecular simulations were generated by using AutoDock Vina (version 1.1.2). The crystal structure of isomaltase from Saccharomyces cerevisiae (PDB ID: 3A4A) was diffusely used in the molecular docking analysis [31]. The 3D structure of compounds **1**-**2** and acarbose was built by ChemBioDraw Ultra 14.0 and energy minimized by ChemBio3D Ultra 14.0 software. The ligands were modified for docking by incorporating nonpolar hydrogen and setting revolvable bonds. As for receptor, all the water molecules were removed and necessary hydrogen atoms were added to the protein. Compounds **1** and **2** and acarbose were considered fully flexible. The grid for docking was formed using *X*-, *Y*-, and *Z*-axes. The docking runs were performed using the Lamarckian genetic algorithm (LGA). In addition, acquiescent arguments were used unless otherwise indicated. The docked formation with the lowest energy value between *α*-glucosidase and active compounds was obtained and visually evaluated using PyMoL 1.7.6 software (http://www.pymol.org/).

### 2.9. Statistical Analysis

The statistical analysis was conducted by the Statistical Program for Social Sciences (SPSS; Chicago, IL) version 13.0 for Windows. All the data were expressed as mean values ± standard deviation (*n* = 3). Statistical significance between groups was assessed by using one-way analysis of variance followed by the LSD test in the condition of variance homogeneity and Dunnett's T3 test in the condition of variance heterogeneity. Differences were considered significant at *p* < 0.05.

## 3. Results and Discussion

### 3.1. Antioxidant and *α*-Glucosidase Inhibitory Activities Evaluation of the Extract and Fractions from Mango Seed Kernel

In this study, the 60% ethanol extract was obtained from the mango seed kernel by ultrasonic extraction, and the extract solution was concentrated *in vacuo*. The crude extract was successively partitioned with petroleum ether, dichloromethane, ethyl acetate, and *n*-butanol. After that, *in vitro* antioxidant and *α*-glucosidase inhibitory activities were evaluated (Table 1). Among the extract and fractions, petroleum ether and dichloromethane fractions showed moderate inhibitory activity against *α*-glucosidase (IC_50_ of 80.10 and 83.58 *μ*g/mL, respectively). The ethyl acetate fraction was found to possess the best antioxidant and *α*-glucosidase inhibitory capacity (IC_50_ of 15.78 and 53.33 *μ*g/mL, respectively). The *n*-butanol fraction showed a positive DPPH radical scavenging activity (IC_50_ of 44.06 *μ*g/mL). Consequently, in order to further clarify the chemical basis in active fractions, we performed additional studies.

### 3.2. GC-MS Analysis of the Bioactive Fraction (the Petroleum Ether Fraction) from Mango Seed Kernel

**Figure 1:**
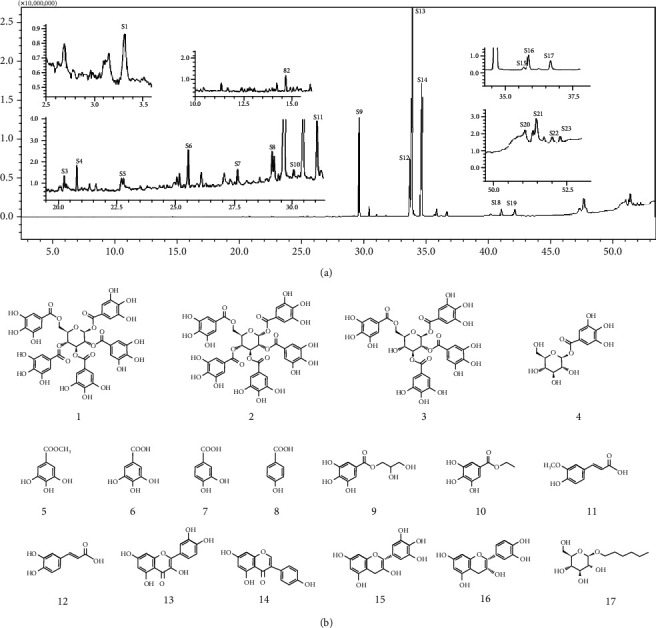
GC-MS chromatogram of the petroleum ether fraction (a) and structures of other active fractions (b) from mango seed kernel.

GC-MS chromatogram of the petroleum ether fraction of mango seed kernel revealed 23 components were detected during retention time 60 min (Figure 1(a)). Mass spectral fingerprint of compounds was confirmed by molecular weight and data library (Table 2). Among all the identified components, eicosane, 9-hexadecenoic acid methyl ester, 9-octadecenoic acid methyl ester, and (*Z*)-3-(heptadec-10-en-1-yl) phenol had never been reported in natural products before and total compounds in the petroleum ether fraction of mango seed kernel were reported for the first time. In addition, specific GC-MS assays also showed that components S9, S12, S13, and S14 were its main ingredients, as reported for this compound making up to 10.3%, 9.31%, 39.11%, and 23.67% of this fraction. The biological activities of main components have been reported in many references. The findings of studies [32, 33] showed that hexadecanoic acid methyl ester, (9*Z*,12*Z*)-9,12-octadecadienoic acid methyl ester, octadecanoic acid methyl ester, and (*E*)-9-octadecenoic acid methyl ester both had significant antioxidant and *α*-glucosidase inhibitory functions. In addition, (9*Z*,12*Z*)-9,12-octadecadienoic acid methyl ester and (*E*)-9-octadecenoic acid methyl ester also showed superior activity against human cancer cell line [34]. Besides this, in the references, the biological activities of other identified components in the petroleum ether fraction of mango seed kernel have been reported. The study of Das, Vasudeva, and Sharma [35] proved that methyl tetradecanoate was a membrane stabilizer and a source of nutrients for protoplasm. Pasquer, Isidore, Zarn, and Keller [36] and Malamy and Klessig [37] suggested that application of 2,4-*bis*(1,1-dimethylethyl)phenol induced expression of PR genes and promoted resistance to bacterial, oomycete, viral, and fungal pathogens in various monocotyledonous and dicotyledonous plants. Meanwhile, the antibacterial effect of 2, 4-*bis*(1,1-dimethylethyl)phenol appeared to correlate with the antioxidant capacity recorded to the compound, as it restrained the production of ROS in both *Phytophthora* and *Aspergillus cinnamomi* [38].

### 3.3. Isolation and Purification of Compounds in Active Fractions from Mango Seed Kernel

Based on the activity results of extract and all fractions, dichloromethane, ethyl acetate, and *n*-butanol fractions were applied to further isolation and purification by chromotography on Sephadex LH-20 column, silica gel column, ODS, and preparative TLC, as well as semipreparative HPLC, et al. Finally, 17 compounds were obtained. Their structures were identified as 1, 2, 3, 4, 6-penta-*O*-galloyl-*β*-D-glucoside (**1**) [14], 1, 2, 3, 4, 6-penta-*O*-galloyl-*α*-D-glucoside (**2**) [15], 1, 2, 3, 6-tetra-*O*-galloyl-*β*-D-glucoside (**3**) [16], 1-*O*-(3, 4, 5-trihydroxybenzoyl)-*β*-D-glucoside (**4**) [17], methyl galate (**5**) [18], gallic acid (**6**) [39], 3,4-dihydroxybenzoic acid (**7**) [19], 4-hydroxybenzoic acid (**8**) [20], 1-glycerol gallate (**9**) [21], ethyl gallate (**10**) (H. [22]), ferulic acid (**11**) [23], caffeic acid (**12**) [24], quercetin (**13**) [25], genistein (**14**) [26], gallocatechin (**15**) [27], epicatechin (**16**) (M. [28]), and hexyl-*β*-D-glucoside (**17**) [29] by comparing their NMR spectroscopic data with data reported in the above references. Compounds **2**, **9**, **15**, and **17** were isolated from the genus *Mangifera* for the first time.

### 3.4. Determination of the DPPH Scavenging Activity of the Isolated Compounds

13 isolated compounds from mango seed kernel were assayed for the antioxidant activity (Table 3). Most of the isolated phenolic compounds showed strong antioxidant activity. The relative order of compounds **1**-**4** in DPPH radical scavenging capacity was found to be compound **1** (IC_50_ = 2.93 ± 1.06 *μ*M) > 2 (IC_50_ = 22.31 ± 0.74 *μ*M) > 3 (IC_50_ = 23.68 ± 0.52 *μ*M) > 4 (IC_50_ = 52.82 ± 0.93 *μ*M) > ascorbic acid (IC_50_ = 116.63 ± 4.16 *μ*M), which suggested that the number of the galloyl moiety played a crucial role in the antioxidant activity, the more it was, the stronger antioxidant activity would be exhibited. Meanwhile, compound **1** showed higher radical scavenging activity than **2**. Hence, compound **1** might have a better spatial configuration than **2** to promote the DPPH radical scavenging activity. The relative order of compounds **5**, **6**, **9**, and **10** in DPPH radical scavenging capacity was found to be compound **5** (IC_50_ = 32.26 ± 0.48 *μ*M) > 6 (IC_50_ = 37.81 ± 2.52 *μ*M) > 10 (IC_50_ = 37.97 ± 1.94 *μ*M) > 9 (IC_50_ > 500 *μ*M), which suggested that the substitution of glycerin at the hydroxyl group of the carboxylic acid in gallic acid significantly reduced DPPH radical scavenging ability. Compound **12** (IC_50_ = 36.91 ± 0.30 *μ*M) exhibited moderate antioxidant activity, which was nearly threefold higher than compound **11** (IC_50_ = 94.99 ± 2.41 *μ*M), respectively. Therefore, the methoxy group at aromatic rings was not beneficial for the radical scavenging activity.

### 3.5. Determination of the *α*-Glucosidase Inhibitory Activity of the Isolated Compounds

*α-Glucosidase* inhibitors could significantly reduce postprandial blood glucose levels in patients with type II diabetes mellitus and reduce diabetic complications. The *α*-glucosidase inhibitory activities of the extract and four fractions had been analyzed above. As shown in Table 3, compounds **2** (IC_50_ = 0.07 ± 0.1 *μ*M) exhibited the strongest inhibitory effect on *α*-glucosidase, which was similar to that of acarbose (IC_50_ = 0.11 ± 0.01 *μ*M), followed by compound **1** (IC_50_ = 0.6 ± 0.36 *μ*M), **3** (IC_50_ = 243.30 ± 1.43 *μ*M), and **4** (IC_50_ > 500 *μ*M). It could be concluded that compound **2** might have a better spatial configuration than **1** to promote the activity, and the amount of the galloyl moiety played a crucial role in *α*-glucosidase inhibition. Compound **6** (IC_50_ = 313.03 ± 3.71 *μ*M) showed low *α*-glucosidase inhibitory activity. Compounds **5**, **9**, and **10** were reported no inhibitory activity. These results indicated that the substitution of methoxyl, ethyoxyl, and glyceryl at the hydroxyl group on the carboxylic acid of gallic acid could reduce the inhibition of *α*-glucosidase. Meanwhile, compound **6** exhibited higher *α*-glucosidase inhibitory potential than **7** (IC_50_ = 425.12 ± 31.50 *μ*M). From this study, it indicated that increasing the number of hydroxyl group on the aromatic ring could enhance the inhibitory activity of *α*-glucosidase.

### 3.6. *α*-Glucosidase Inhibition Kinetics of Compounds **1** and **2**

**Figure 2:**
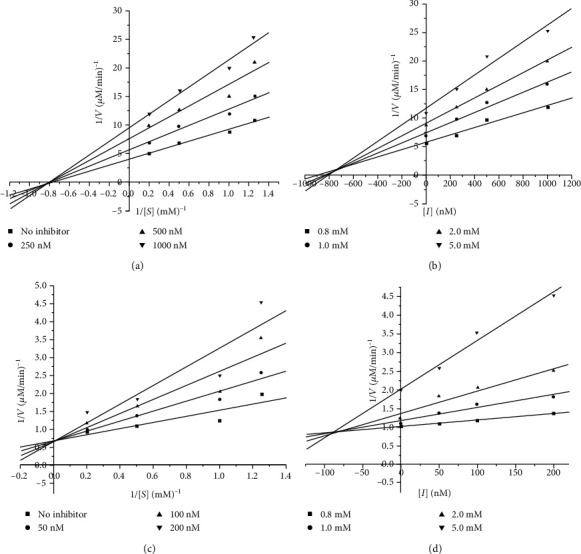
Lineweaver-Burk plots and Dixon plots for the inhibition of *α*-glucosidase by compounds 1 (a, b) and 2 (c, d).

Compounds **1** and **2** both had strong inhibitory activities on *α*-glycosidase, especially **2**. Therefore, it was necessary to investigate the inhibition types and calculate the inhibition kinetics constants (*K*_i_). Results of enzyme kinetics study were performed by using Dixon plots and Lineweaver-Burk plots. The results were displayed in Table 3 and Figure 2. In Figure 2, *K*_m_ values of *α*-glucosidase in the existence of **1** did not change but *V*_max_ values of *α*-glucosidase with the compounds progressively reduced, pointing that **1** was in a noncompetitive inhibitor manner with the *K*_i_ value of 732.95 ± 0.22 nM. In addition, **2** restrained *α*-glucosidase in competitive manner as the *V*_max_ maintained constant and *K*_m_ improved. Meanwhile, *K*_i_ value of this compound was calculated as 98.37 ± 0.31 nM. Competitive inhibitors were known to compete with substrates for the active sites of enzymes, whereas noncompetitive inhibitors primarily bind to allosteric sites of enzyme-substrate complexes [40].

### 3.7. Molecular Docking Studies

**Figure 3:**
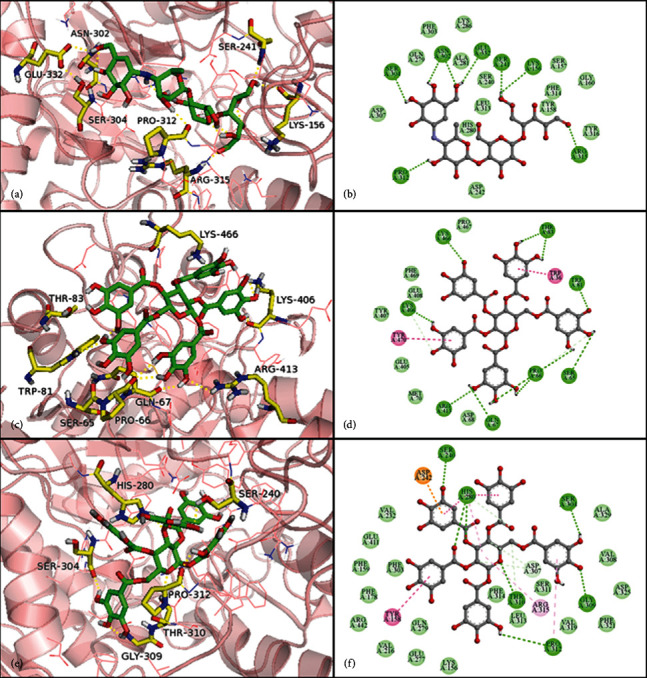
The binding modes and 2D diagram of *α*-glucosidase inhibition by acarbose (a, b), 1, 2, 3, 4, 6-Penta-*O*-galloyl-*β*-D-glucoside (1) (c, d) and 1, 2, 3, 4, 6-Penta-*O*-galloyl-*α*-D-glucoside (2) (e, f). The small molecules were shown in green sticks, while the hydrogen bonds were shown in yellow sticks (For interpretation of the references to color in this figure legend, the reader is referred to the web version of this article.).

In order to understand how isolated compounds **1** and **2** conjugate with *α*-glucosidase, molecular docking was implemented. Because of the existence of the galloyl moiety, compound **2** reached deeper the active pocket than acarbose (Figure 3). In the interaction of compound **2** with *α*-glucosidase, **2** was encompassed by the residues VAL-232, PHE-303, PHE-178, VAL-216, GLN279, LYS-156, LEU-313, ASP-307, PHE-321, VAL-308, and ALA-329. In addition, some active hydroxyl groups were involved in the formation of hydrogen bonds, such as the galloyl at the 3-OH of sugar moiety at the C-1 and C-6 with side chains of PRO-312 and SER-304, respectively, the galloyl at the 3-O on sugar moiety at the C-3 position and the galloyl at the 4-O on sugar moiety at the C-6 position with side chains of SER-240 and GLY-309, and the carbonyl group on the galloyl on sugar moiety at the C-2 and oxygen atom on the sugar moiety with HIS-280 and THR-310, respectively. Meanwhile, *π*-*π* T-shaped, *π*-alkyl, and electrostatic interactions facilitated the tight bonding between the enzyme and compound **2**. By contrast, acarbose could only form seven hydrogen bonds.

To investigate the allosteric site of the *α*-glucosidase bond with noncompetitive inhibitors (**1**), we implemented blind docking by establishing a grid involved in all enzymes. Compound **1** was surrounded by a number of catalytic amino acid residues (Figure 3), including PRO-467, PHE-469, GLU-408, MET-70, and ASP-68. Eight hydrogen bonds were present between the galloyl at the 1, 2, 3-OH and 1-O on sugar moiety at the C-1 position, the galloyl at the 1-OH on sugar moiety at the C-2 position, the galloyl at the 1-O on sugar moiety at the C-3 position, the galloyl at the 3, 4-OH on sugar moiety at the C-4 position and the galloyl at the 3, 4-OH, and 5-O on sugar moiety at the C-6 position of compound **1** with GLN-67, PRO-66, ARG-413, LYS-406, LYS-466, THR-83, TRP-81, and SER-65, respectively. The aromatic rings of the galloyl on sugar moiety at the C-4 and C-2 positions of compound **1** formed *π*-*π* T-shaped and *π*-*π* stacked interactions with TRP-36 and TYR-470, respectively.

In recent years, molecular docking simulations have become an important tool for understanding the interaction mode and the structure-activity relationships of ligands with receptors. The in silico research results of compounds **1** and **2** were in agreement with the IC_50_ data and the enzyme kinetic study *in vitro*. The integration of enzyme activity, kinetics, and molecular docking studies provided principle insights into the molecular basis underlying ligand binding affinity and *α*-glucosidase inhibition.

## 4. Conclusions

In summary, antioxidant and *α*-glucosidase inhibitory activities of the extract, fractions, and isolated compounds from mango seed kernel were first documented. The petroleum ether, dichloromethane, ethyl acetate, and *n*-butanol fractions exhibited different degrees of antioxidant and *α*-glucosidase inhibitory activity. In order to further clarify the chemical basis, a total of 23 ingredients were identified by efficient and fast GC-MS analysis from the petroleum ether fraction. As far as we know, it was the first report on the detailed compounds of the petroleum ether fraction of mango seed kernel. Among all the identified components, (*Z*)-3-(Heptadec-10-en-1-yl) phenol, eicosane, 9-octadecenoic acid methyl ester, and 9-hexadecenoic acid methyl ester had never been reported before. In addition, 17 compounds were isolated and identified from other active fractions of mango seed kernel. 1, 2, 3, 4, 6-penta-*O*-galloyl-*α*-D-glucoside (**2**), 1-glycerol gallate (**9**), gallocatechin (**15**), and hexyl-*β*-D-glucoside (**17**) were isolated from *Mangifera* for the first time. Among isolated components, 1, 2, 3, 4, 6-penta-*O*-galloyl-*β*-D-glucoside (**1**) and 1, 2, 3, 4, 6-penta-*O*-galloyl-*α*-D-glucoside (**2**) showed stronger DPPH radical scavenging and *α*-glucosidase inhibitory capacities than others. The structure-activity relationship has been discussed. According to the kinetic research, **2** inhibited *α*-glucosidase in a competitive manner, whereas **1** acted in a noncompetitive manner. Molecular docking studies showed that compound **1** accommodated well in the allosteric site of the *α*-glucosidase interacting with a number of crucial amino residues. Compound **2** reached deeper the active site pocket of *α*-glucosidase than acarbose. The stability of binding was enhanced by *π*-*π* T-shaped, *π*-alkyl, hydrogen bond, and electrostatic interactions. These results might be useful to investigate the pharmacological activity in this fruit. Meanwhile, our study provided a chemical basis for the further development and utilization of mango seed kernel in the food and medicine fields.

## Figures and Tables

**Table 1:** Antioxidant and *α*-glucosidase inhibitory activities of the 60% ethanol extract and fractions from mango seed kernel.

Extract and fractions	DPPH assay^B^	*α*-Glucosidase inhibitory^B^
	IC_50_^A^ (*μ*g/mL)	IC_50_^A^ (*μ*g/mL)
60% ethanol extract	206.42 ± 3.97^b^	170.46 ± 38.56^a^
Petroleum ether fraction	>1000	80.10 ± 20.74^d^
Dichloromethane fraction	362.32 ± 17.47^a^	83.58 ± 7.78^c^
Ethyl acetate fraction	15.78 ± 0.90^d^	53.33 ± 1.89^e^
*n*-Butanol fraction	44.06 ± 0.60^c^	100.39 ± 8.71^b^
Ascorbic acid^C^	9.37 ± 0.84^e^	—
Acarbose^D^	—	50.90 ± 4.11^f^

^A^Data were represented as the mean value ± SD, *n* = 3. Values followed by different letters are significantly different (*p* ≤ 0.05). ^B^The test concentrations ranged from 62.5 to 1000 *μ*g/mL. ^C^Positive control (DPPH assay). ^D^Positive control (*α*-glucosidase inhibitory effect).

**Table 2:** Phytochemical components of the petroleum ether fraction of mango seed kernel using GC-MS.

Components	Retention time (min)	Molecular formula	Chemical structure inferred by NIST14 and NIST14s	Compound name	Integrated peak area	Relative contents (%)
S1	3.32	C_8_H_10_	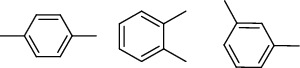	1,4-Dimethylbenzene, 1,2-Dimethylbenzene, 1,3-Dimethylbenzene	61583	0.02
S2	14.23	C_15_H_32_	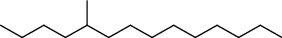	5-Methyltetradecane	113842	0.03
S3	20.27	C_20_H_42_	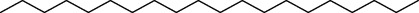	Eicosane	145274	0.04
S4	20.81	C_14_H_22_O	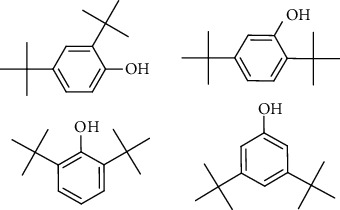	2,4-*Bis*(1,1-dimethylethyl)phenol, 2,5-*bis*(1,1-dimethylethyl)phenol, 2,6-*bis*(1,1-dimethylethyl)phenol, 3,5-*bis*(1,1-dimethylethyl)phenol	353443	0.09
S5	22.81	C_12_H_26_O_2_	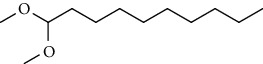	Decanal dimethyl acetal	85855	0.02
S6	25.56	C_15_H_30_O_2_	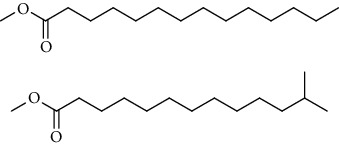	Methyl tetradecanoate, 12-methyl-tridecanoic acid methyl ester	490546	0.13
S7	27.67	C_16_H_32_O_2_	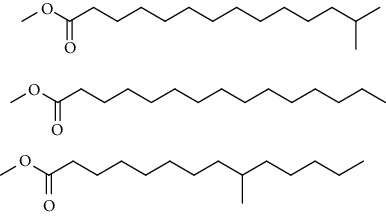	Methyl 13-methyltetradecanoate, pentadecanoic acid methyl ester, methyl 9-methyltetradecanoate	123499	0.03
S8	29.27	C_17_H_32_O_2_	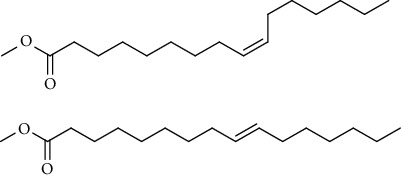	(Z)-9-hexadecenoic acid methyl ester (E)-9-hexadecenoic acid methyl ester......................	312468	0.08
S9	29.68	C_17_H_34_O_2_	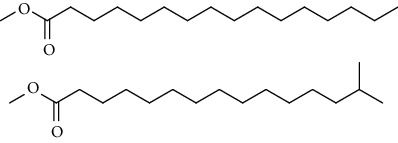	Hexadecanoic acid methyl ester, methyl 14-methylpentadecanoate	39719527	10.30
S10	30.10	C_18_H_28_O_3_	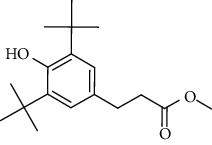	3,5-*Bis*(1,1-dimethylethyl) Benzene propanoic acid methyl ester	73620	0.02
S11	31.08	C_18_H_36_O_2_	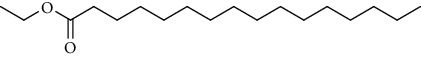	Hexadecanoic acid ethyl ester	1650498	0.43
S12	33.73	C_19_H_34_O_2_	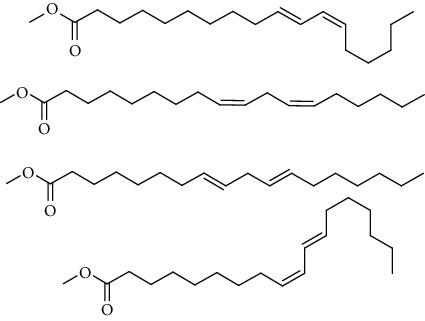	(10*E*,12*Z*)-10,12-octadecadienoic acid methyl ester, (9*Z*,12*Z*)-9,12-octadecadienoic acid methyl ester, (8*E*,11*E*)-8,11-octadecadienoic acid methyl ester, (9*Z*,11*E*)-9,11-octadecadienoic acid methyl ester	35910705	9.31
S13	34.07	C_19_H_36_O_2_	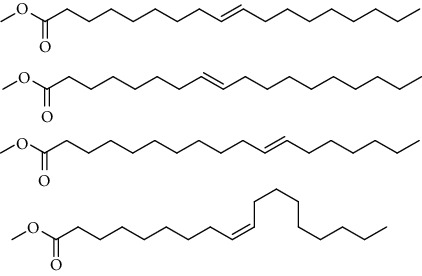	(E)-9-octadecenoic acid methyl ester, (E)-8-octadecenoic acid methyl ester, (E)-11-octadecenoic acid methyl ester, (Z)-9-octadecenoic acid methyl ester	150909725	39.11
S14	34.67	C_19_H_38_O_2_	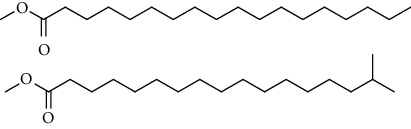	Octadecanoic acid methyl ester, 16-methyl-heptadecanoic acid methyl ester	91261893	23.67
S15	35.72	C_20_H_36_O_2_	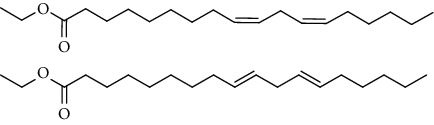	Linoleic acid ethyl ester, (9*E*,12*E*)-9,12-octadecadienoic acid ethyl ester	804755	0.21
S16	35.89	C_20_H_38_O_2_	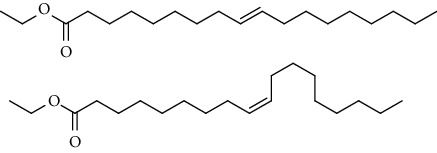	(E)-9-octadecenoic acid ethyl ester, (Z)-9-octadecenoic acid ethyl ester	4489705	1.16
S17	36.70	C_20_H_40_O_2_	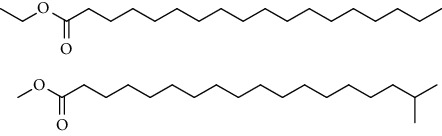	Octadecanoic acid ethyl ester, 17-methyl-octadecanoic acid methyl ester	2894869	0.75
S18	41.07	C_21_H_42_O_2_	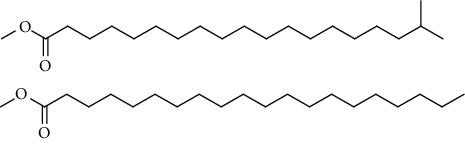	Methyl 18-methylnonadecanoate, eicosanoic acid methyl ester	4768435	1.24
S19	42.23	C_18_H_35_NO	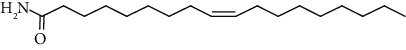	(Z)-9-octadecenamide	4999183	1.30
S20	51.10	C_23_H_36_O	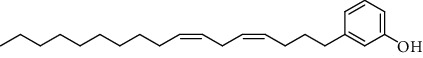	3-((4*Z*,7*Z*)-heptadeca-4,7-dien-1-yl)phenol	1057168	0.27
S21	51.36	C_23_H_38_O	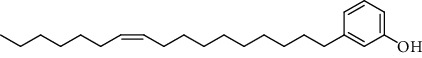	(Z)-3-(heptadec-10-en-1-yl)phenol	5704580	1.48
S22	52.02	C_21_H_42_O_4_	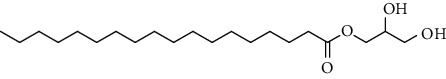	Octadecanoic acid, 2,3-dihydroxypropyl ester	783622	0.20
S23	52.30	C_25_H_50_O_2_	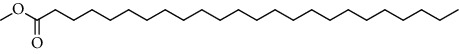	Tetracosanoic acid methyl ester	1027137	0.27

**Table 3:** Antioxidant and *α*-glucosidase inhibitory activities of the isolated compounds from mango seed kernel.

Compounds	DPPH assay^B^	*α*-Glucosidase inhibitory^C^		
	IC_50_^A^ (*μ*M)	IC_50_^A^ (*μ*M)	Inhibition mode^D^	*K*_i_ (nM)^E^
1	2.93 ± 1.06^j^	0.60 ± 0.36^e^	Noncompetitive	732.95 ± 0.22
2	22.31 ± 0.74^i^	0.07 ± 0.10^e^	Competitive	98.37 ± 0.31
3	23.68 ± 0.52^i^	243.30 ± 1.43^c^	—	—
4	52.82 ± 0.93^d^	>500	—	—
5	32.26 ± 0.48^h^	>500	—	—
6	37.81 ± 2.52^f^	313.03 ± 3.71^b^	—	—
7	38.19 ± 1.03^f^	425.12 ± 31.50^a^	—	—
9	>500	>500	—	—
10	37.97 ± 1.94^f^	>500	—	—
11	94.99 ± 2.41^b^	>500	—	—
12	36.91 ± 0.30^g^	>500	—	—
13	59.97 ± 0.82^c^	137.94 ± 7.07^d^	—	—
16	40.15 ± 0.82^e^	>500	—	—
Ascorbic acid^F^	116.63 ± 4.16^a^	—	—	—
Acarbose^G^	—	0.11 ± 0.01^e^	—	—

^A^Data were represented as the mean value ± SD, 6. Values followed by different letters are significantly different (*p* ≤ 0.05). ^B^The test concentrations ranged from 15.625 to 500 *μ*M. ^C^The test concentrations ranged from 125 to 2000 *μ*M. ^D^Determined by Lineweaver-Burk plots. ^E^Determined by Dixon plots. ^F^Positive control (DPPH assay). ^G^Positive control (*α*-glucosidase inhibitory effect).

## Data Availability

The underlying data supporting our findings can be found and generated during the study.

## Supplementary Materials

Supplementary Materials Figure S1: GC-MS chromatogram of the petroleum ether fraction from mango kernel. Figure S2 to Figure S24: GC-MS spectrum of component 1 to 23. Table S1: molecular interactions of the *α*-glucosidase (3A4A) active site with compounds 1 and 2.
