# Supplementary material for: Antioxidant and α-Glucosidase Inhibitory Activities Guided Isolation and Identification of Components from Mango Seed Kernel
Source: Oxid Med Cell Longev. 2020 Dec 28;2020:8858578. doi: 10.1155/2020/8858578 (PMC7785352; doi:10.1155/2020/8858578)
Supplement: Supplementary Materials — Figure S1: GC-MS chromatogram of the petroleum ether fraction from mango kernel. Figure S2 to Figure S24: GC-MS spectrum of component 1 to 23. Table S1: molecular interactions of the α-glucosidase (3A4A) active site with compounds 1 and 2. [file 8858578.f1.pdf]

Antioxidant and α-glucosidase inhibitory activities guided isolation and identification of components from mango seed kernel

Dan Yang[a,1], Xuee Chen[a,1], Xida Liu[a], Na Han[a], Zhihui Liu[a], Sikai Li[a], Jianxiu Zhai[a], Jun Yin[a*]

[a] *Department of Pharmacognosy and Utilization Key Laboratory of Northeast Plant Materials, School of Traditional Chinese Medicine, Shenyang Pharmaceutical University, Shenyang 110016, China*

*Corresponding author

Address: Shenyang Pharmaceutical University, 103 Wenhua Road, Shenhe district, China.

E-mail address: 362354426@qq.com (Dan Yang), cxe1210@163.com (Xuee Chen), 441590920@qq.com (Xida Liu), hanna82@163.com (Na Han), liuzhihuishenyang@163.com (Zhihui Liu), lihaoshuo672406281@126.com (Sikai Li), lookingformilk@163.com (Jianxiu Zhai), yinjun826@sina.com (Jun Yin)

[1] These authors are joint the first authors.

List for supporting information

Figure S1. GC–MS chromatogram of the petroleum ether fraction from mango kernel............3
Figure S2. GC-MS spectrum of component **S1**..4
Figure S3. GC-MS spectrum of component **S2**..4
Figure S4. GC-MS spectrum of component **S3**..4
Figure S5. GC-MS spectrum of component **S4**..5
Figure S6. GC-MS spectrum of component **S5**..5
Figure S7. GC-MS spectrum of component **S6**..6
Figure S8. GC-MS spectrum of component **S7**..6
Figure S9. GC-MS spectrum of component **S8**..7
Figure S10. GC-MS spectrum of component **S9**..7
Figure S11. GC-MS spectrum of component **S10**..7
Figure S12. GC-MS spectrum of component **S11**..8
Figure S13. GC-MS spectrum of component **S12**..8
Figure S14. GC-MS spectrum of component **S13**..9
Figure S15. GC-MS spectrum of component **S14**..9
Figure S16. GC-MS spectrum of component **S15**..10
Figure S17. GC-MS spectrum of component **S16**..10
Figure S18. GC-MS spectrum of component **S17**..10
Figure S19. GC-MS spectrum of component **S18**..11
Figure S20. GC-MS spectrum of component **S19**..11
Figure S21. GC-MS spectrum of component **S20**..11
Figure S22. GC-MS spectrum of component **S21**..12
Figure S23. GC-MS spectrum of component **S22**..12
Figure S24. GC-MS spectrum of component **S23**..12
Table S1 Molecular interactions of the α-glucosidase (3A4A) active site with compounds **1-2**..13

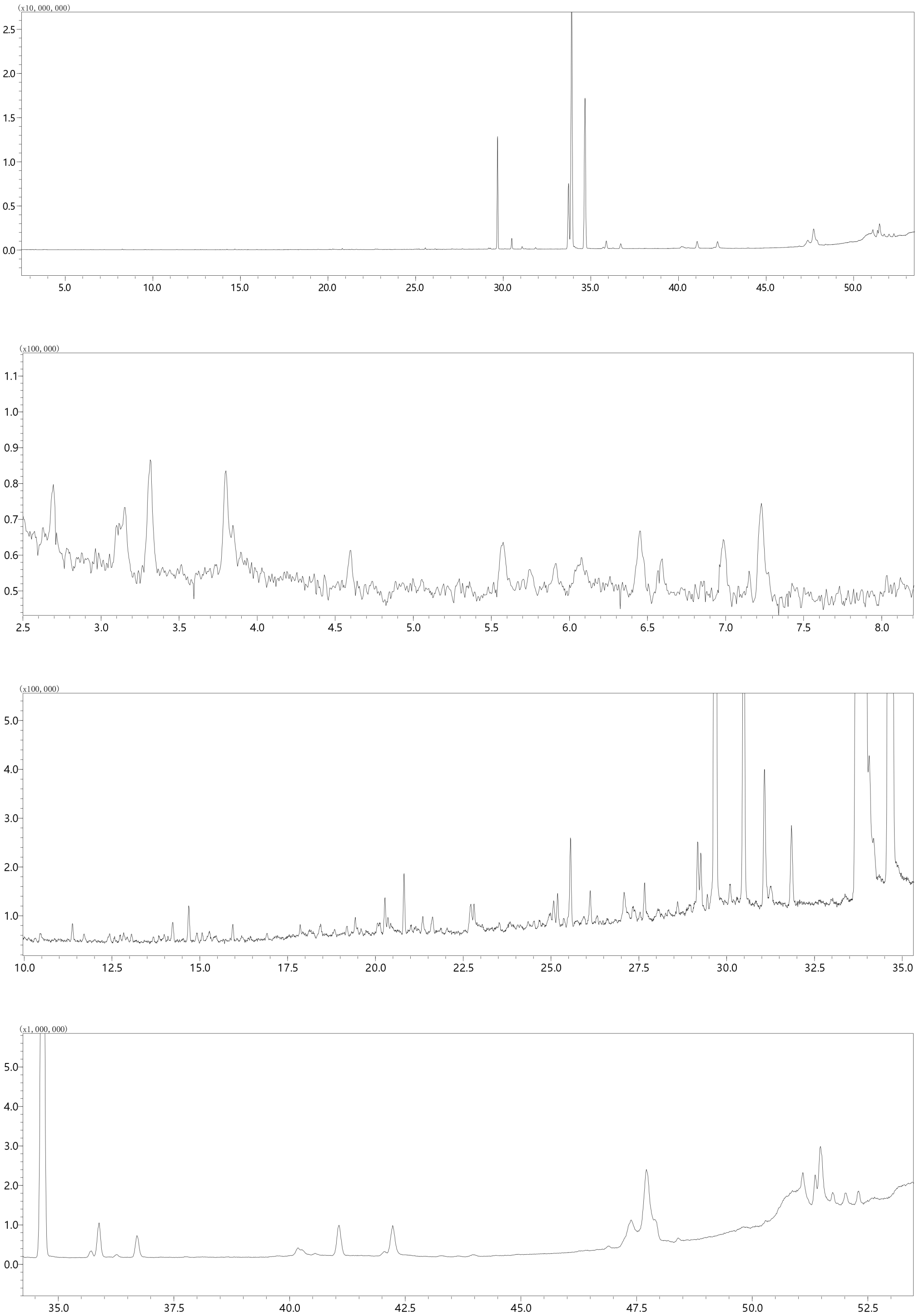

Figure S1. GC–MS chromatogram of the petroleum ether fraction from mango seed kernel

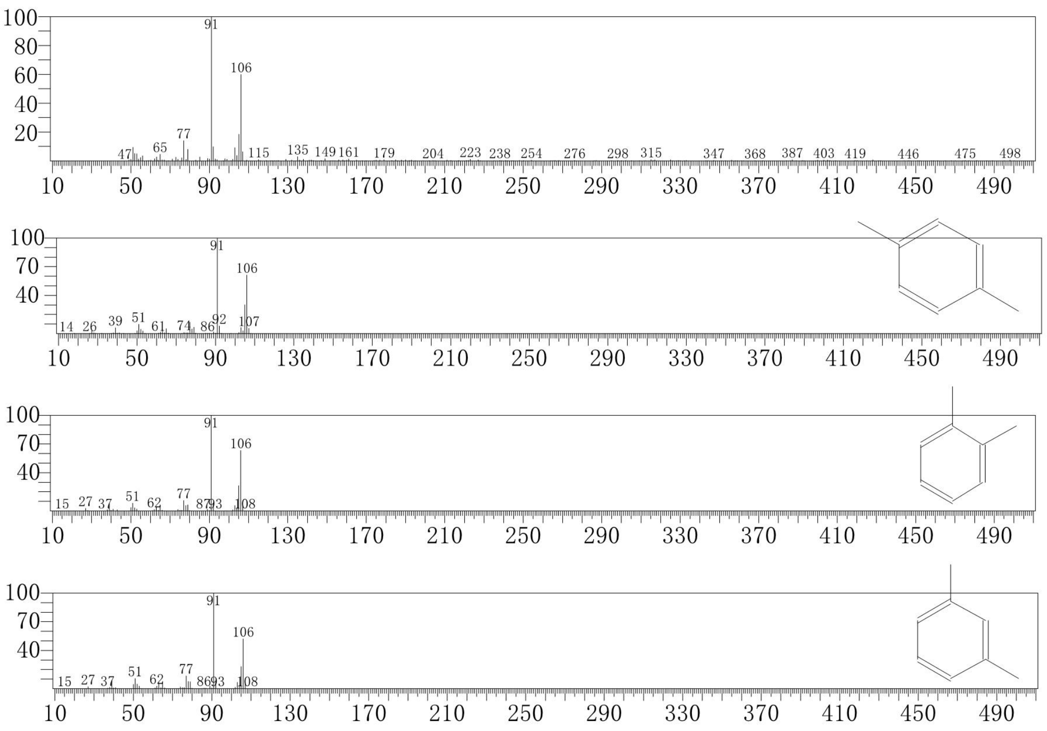

Figure S2. GC-MS spectrum of component **1**

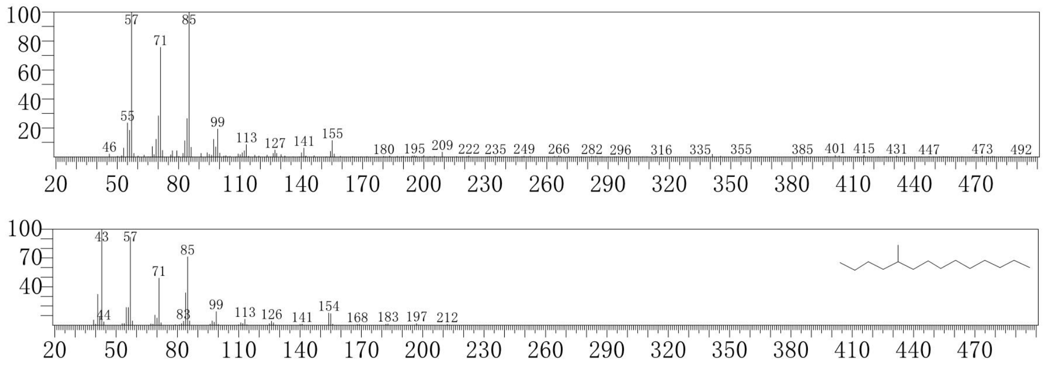

Figure S3. GC-MS spectrum of component **2**

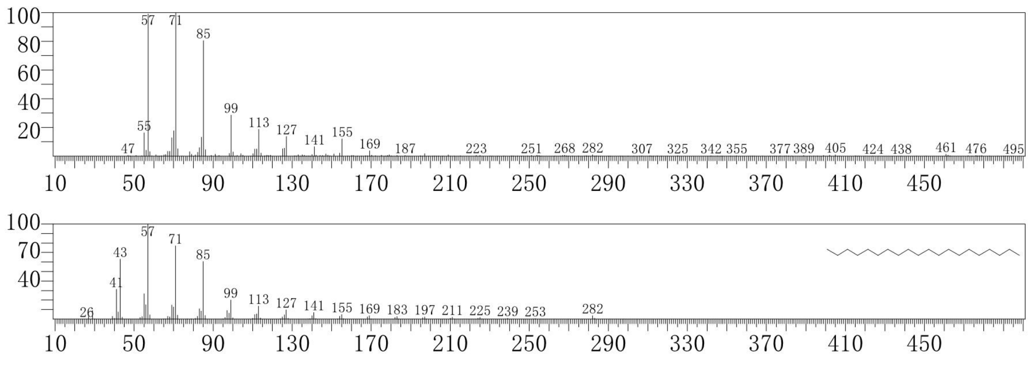

Figure S4. GC-MS spectrum of component **3**

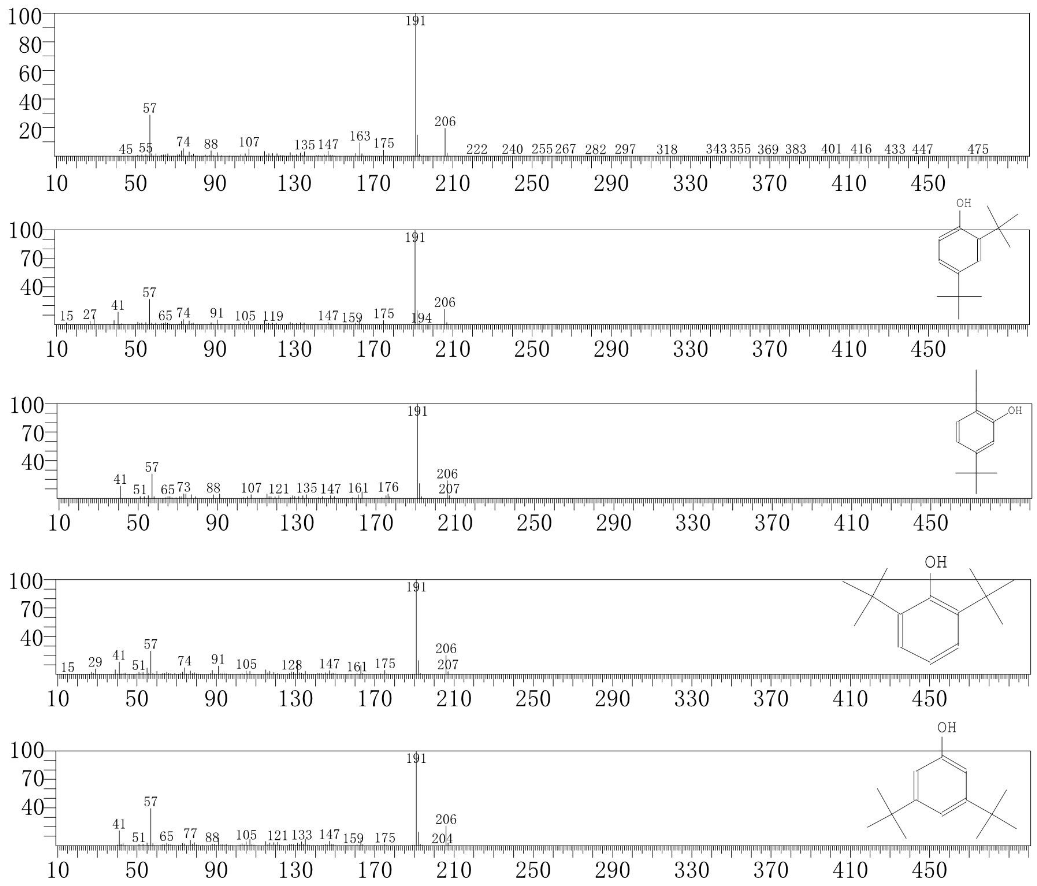

Figure S5. GC-MS spectrum of component **4**

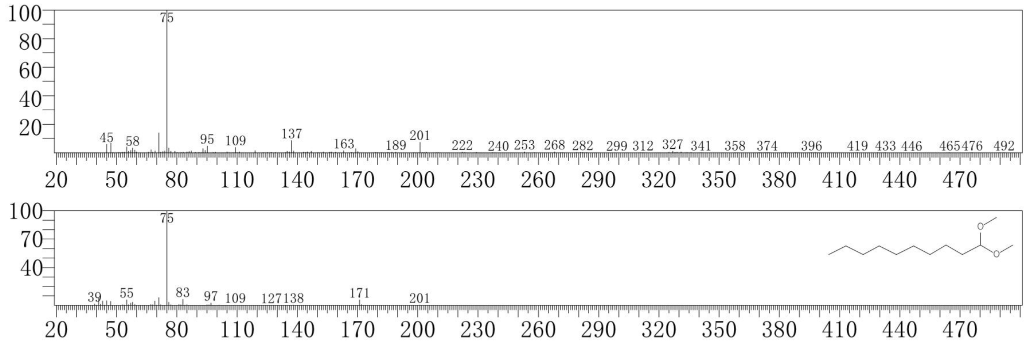

Figure S6. GC-MS spectrum of component **5**

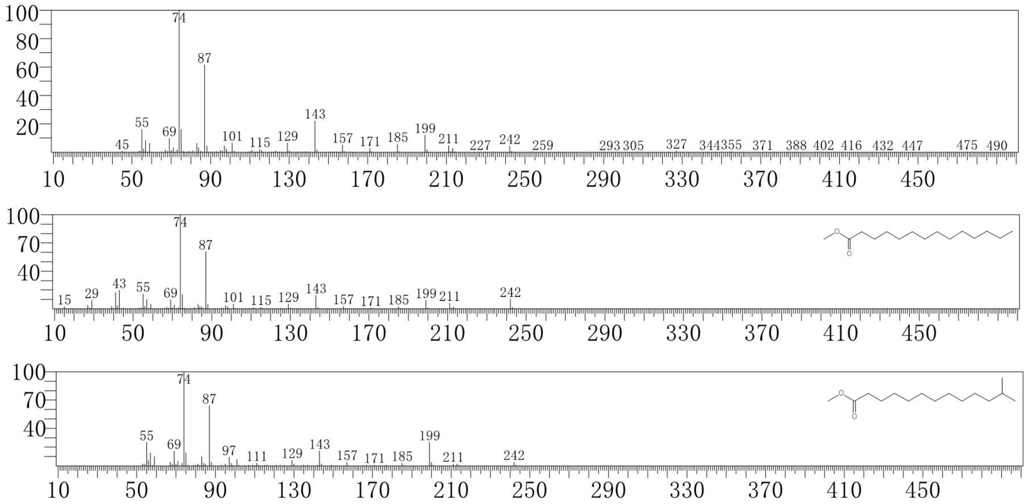

Figure S7. GC-MS spectrum of component **6**

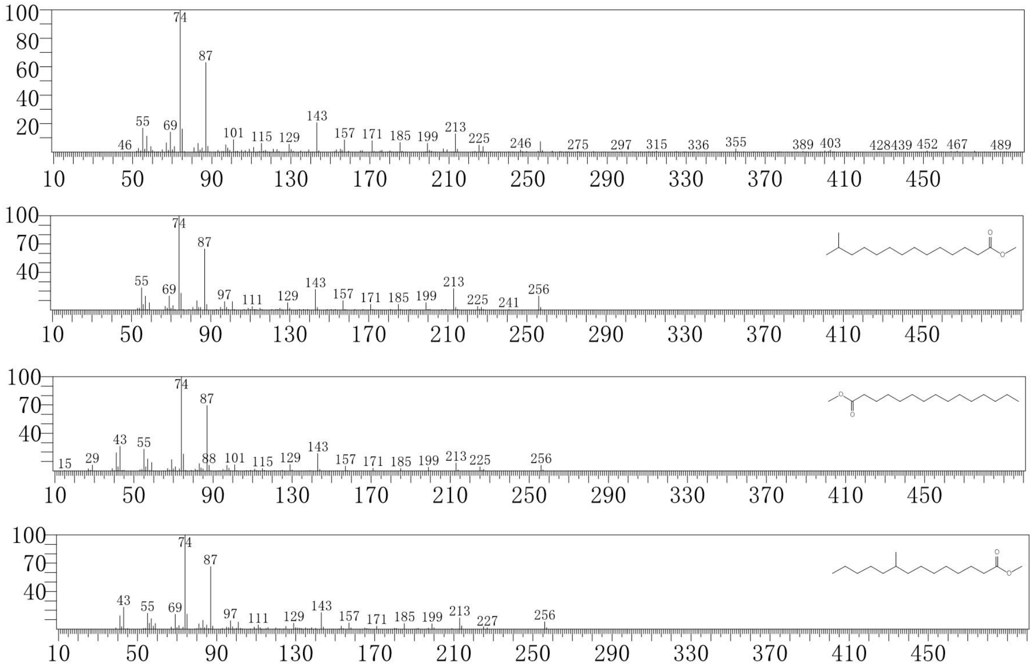

Figure S8. GC-MS spectrum of component **7**

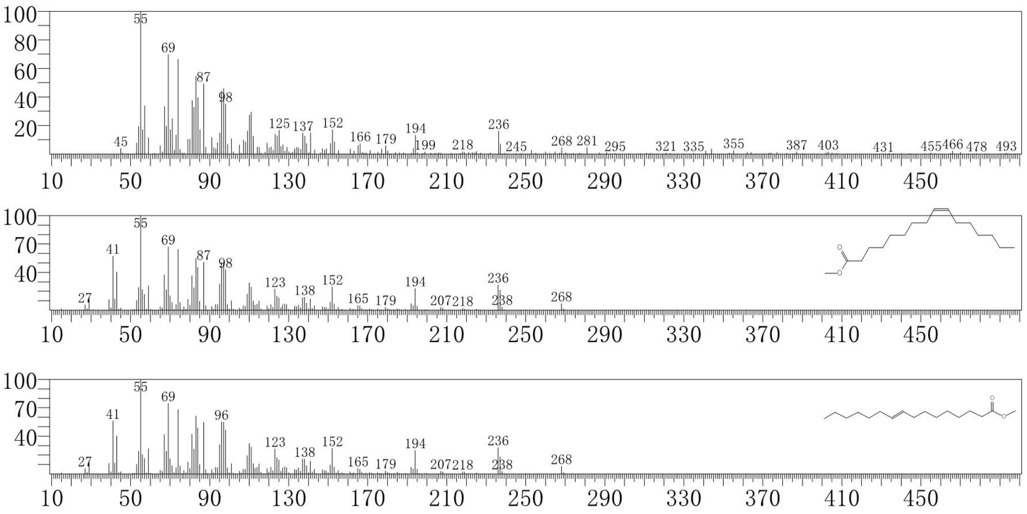

Figure S9. GC-MS spectrum of component **8**

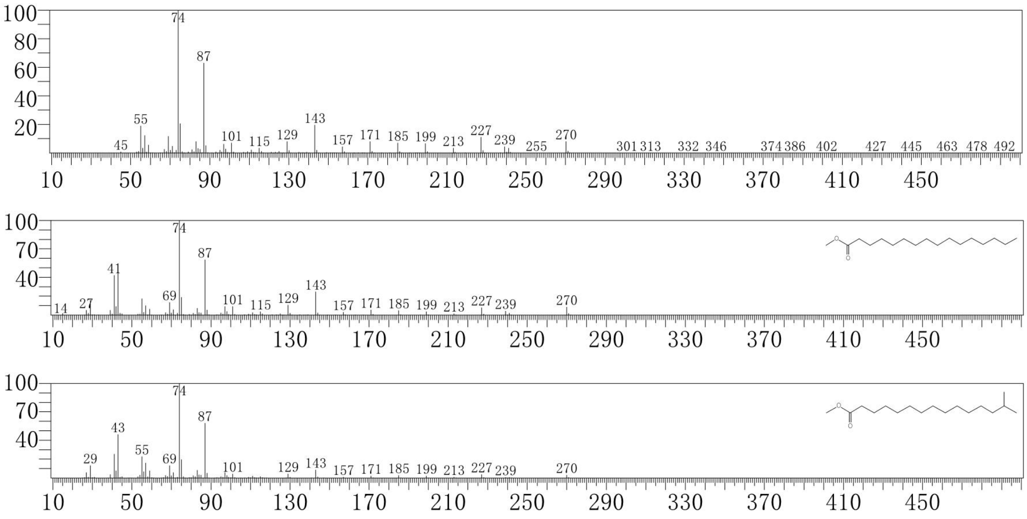

Figure S10. GC-MS spectrum of component **9**

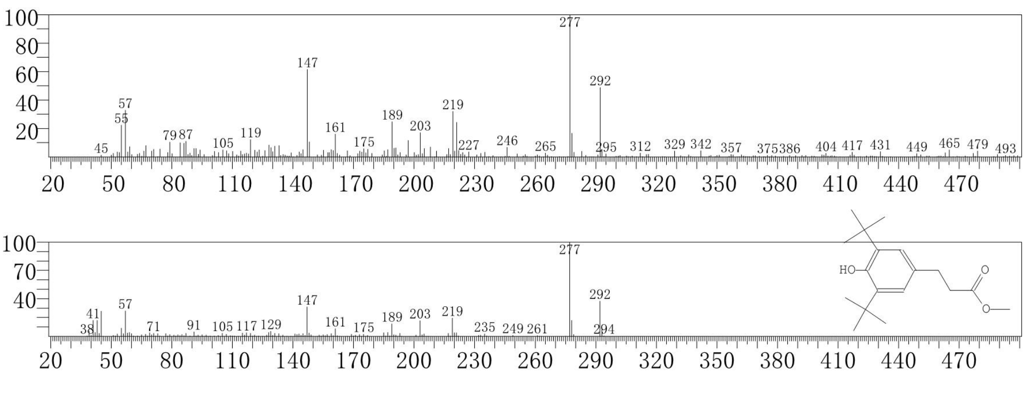

Figure S11. GC-MS spectrum of component **10**

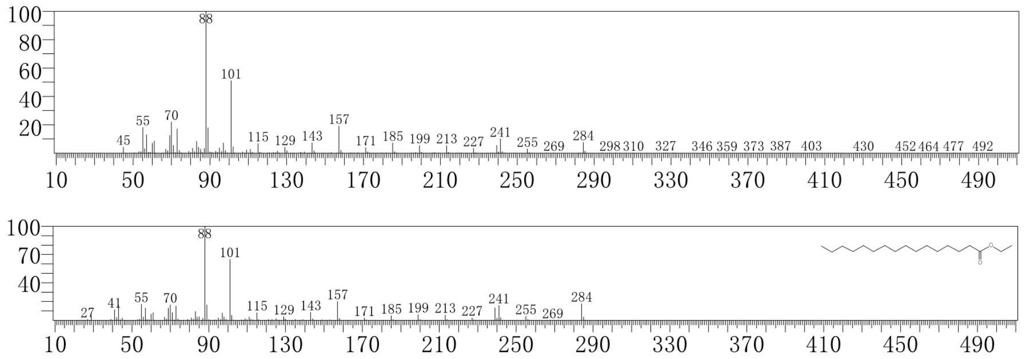

Figure S12. GC-MS spectrum of component **11**

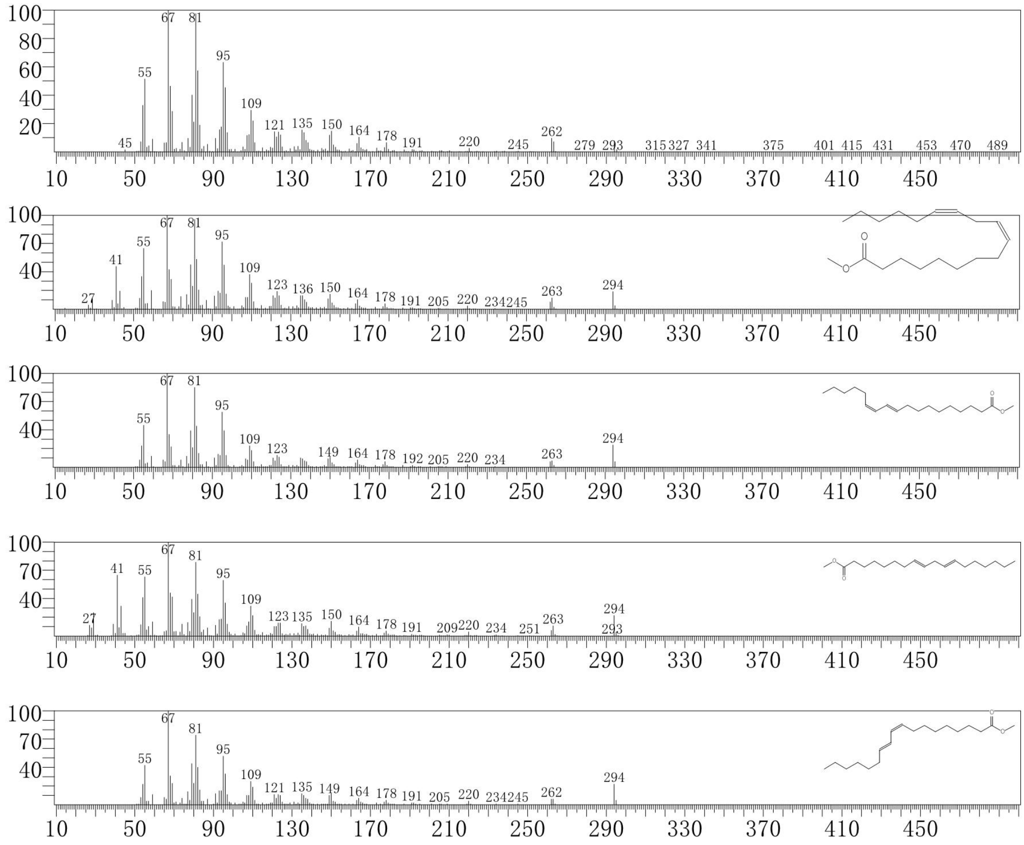

Figure S13. GC-MS spectrum of component **12**

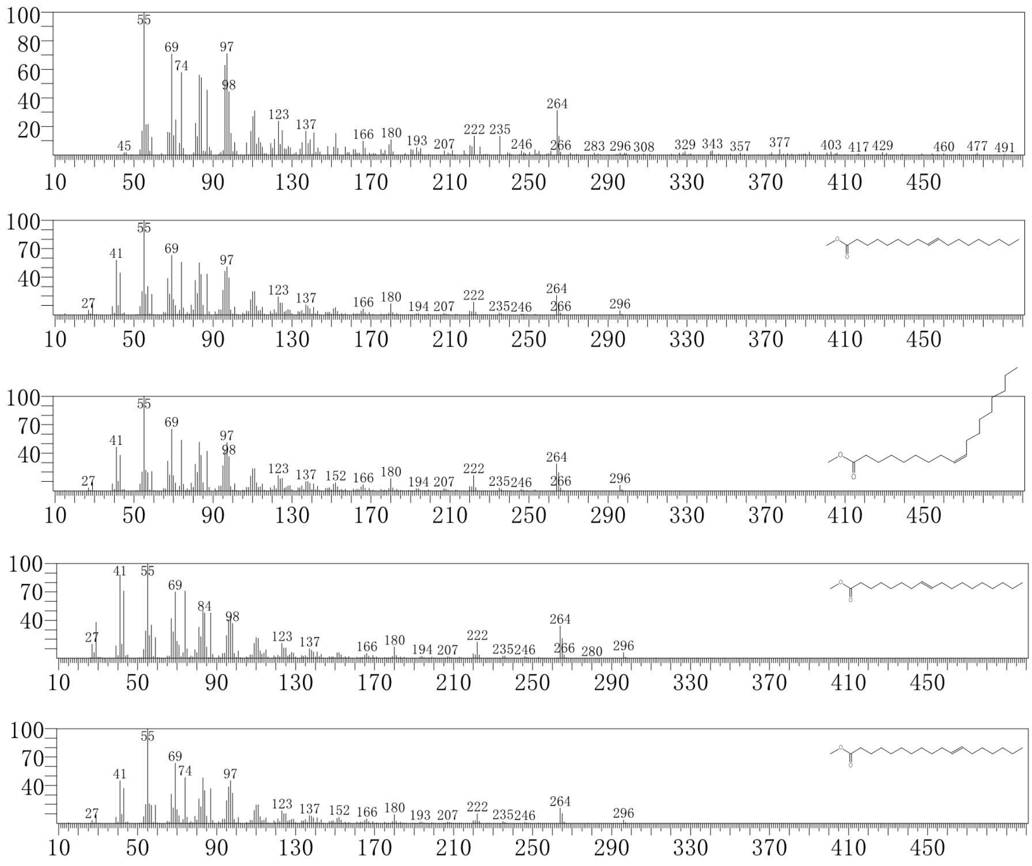

Figure S14. GC-MS spectrum of component **13**

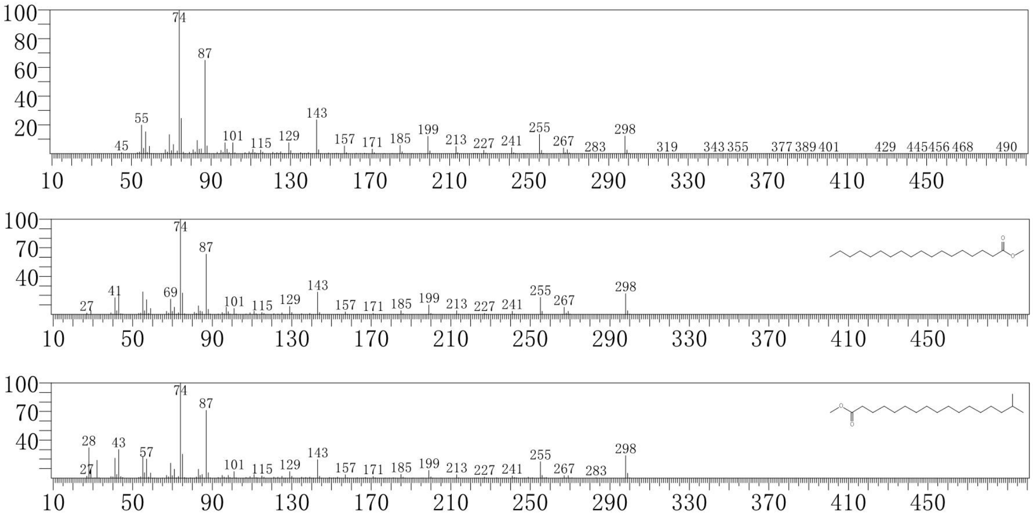

Figure S15. GC-MS spectrum of component **14**

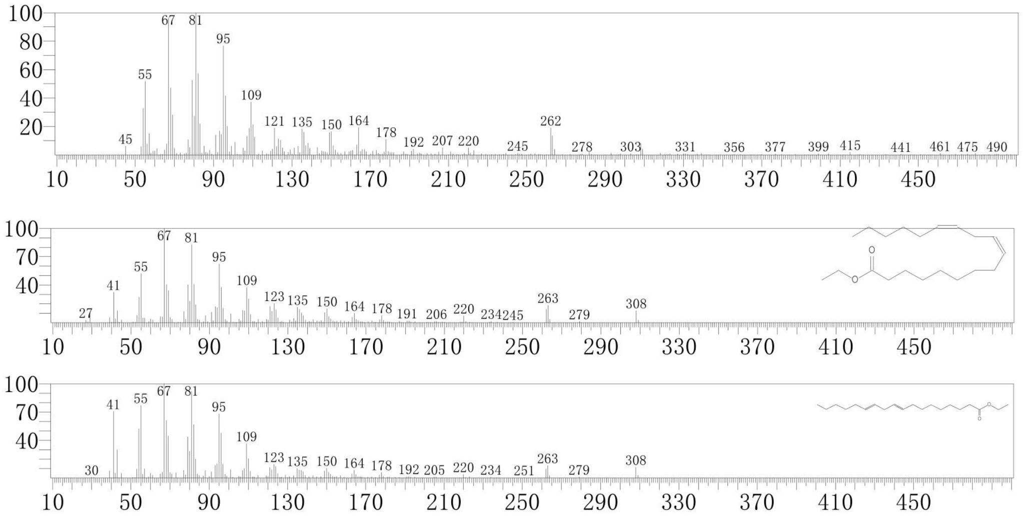

Figure S16. GC-MS spectrum of component **15**

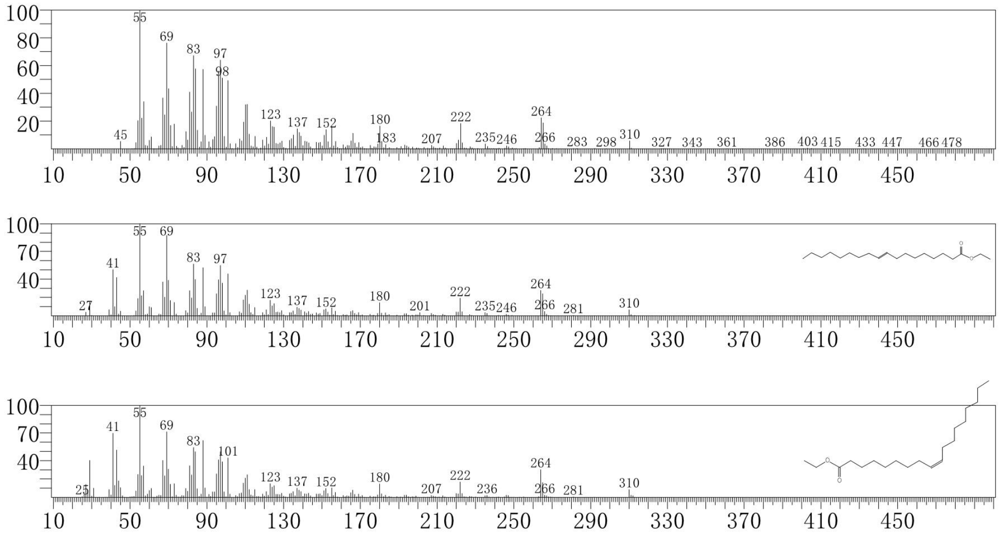

Figure S17. GC-MS spectrum of component **16**

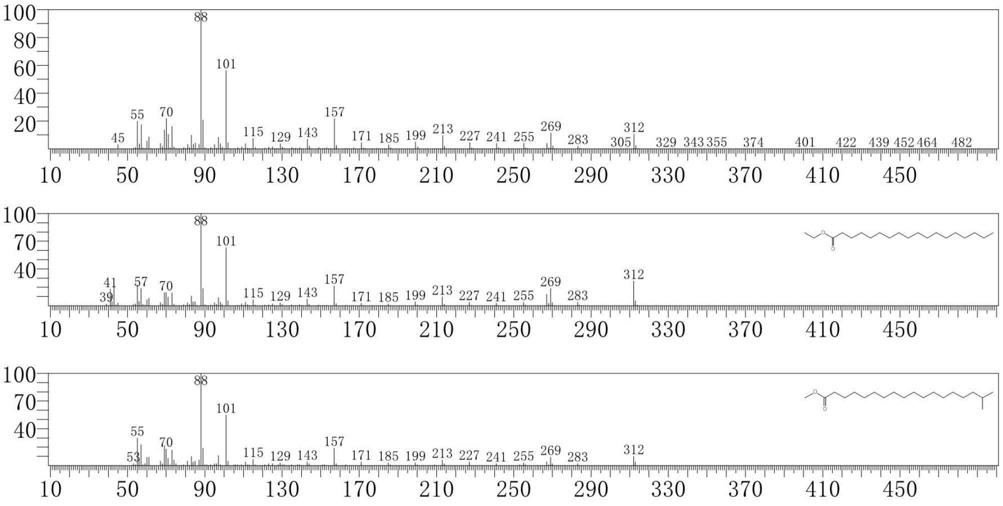

Figure S18. GC-MS spectrum of component **17**

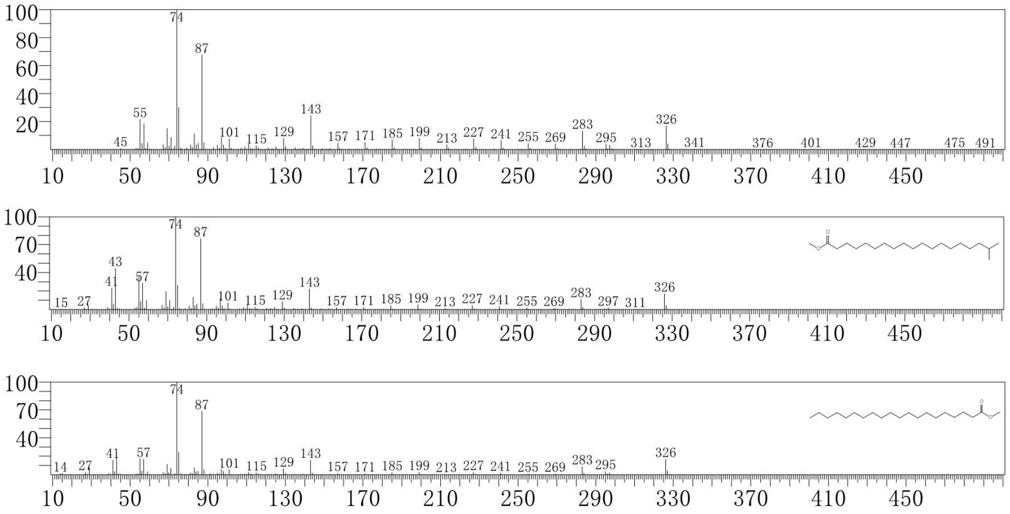

Figure S19. GC-MS spectrum of component **18**

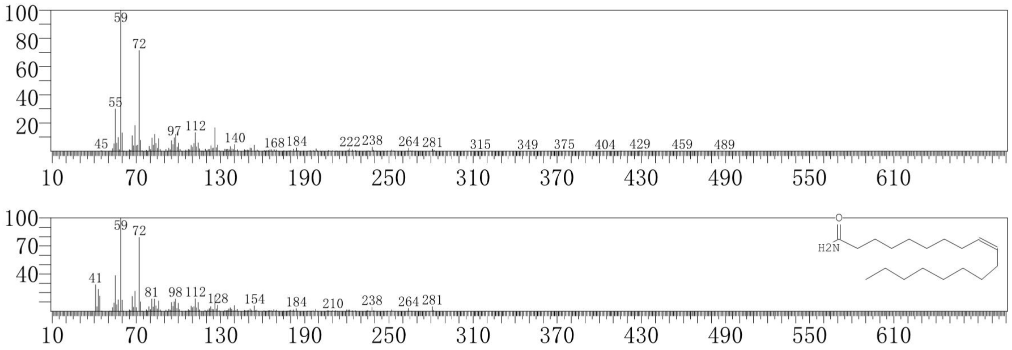

Figure S20. GC-MS spectrum of component **19**

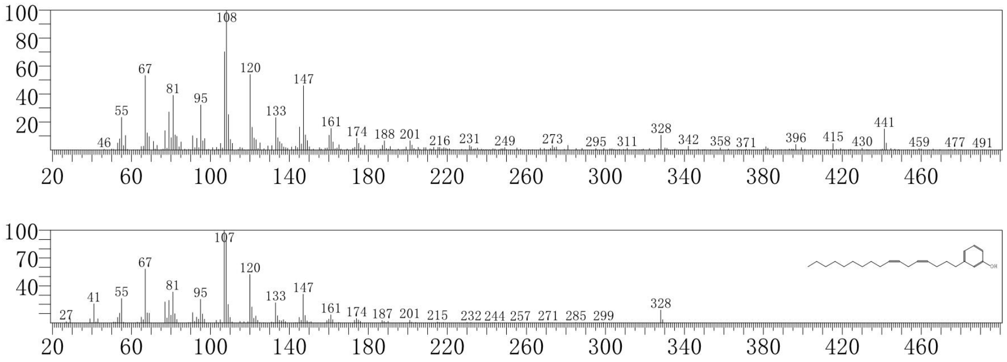

Figure S21. GC-MS spectrum of component **20**

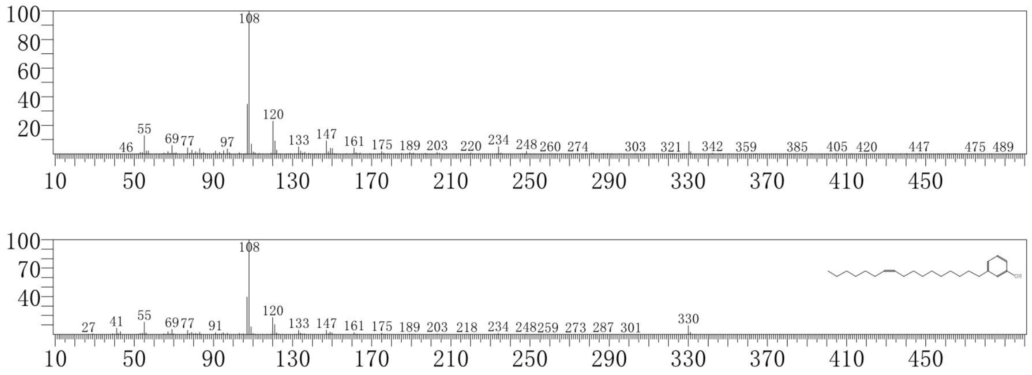

Figure S22. GC-MS spectrum of component **21**

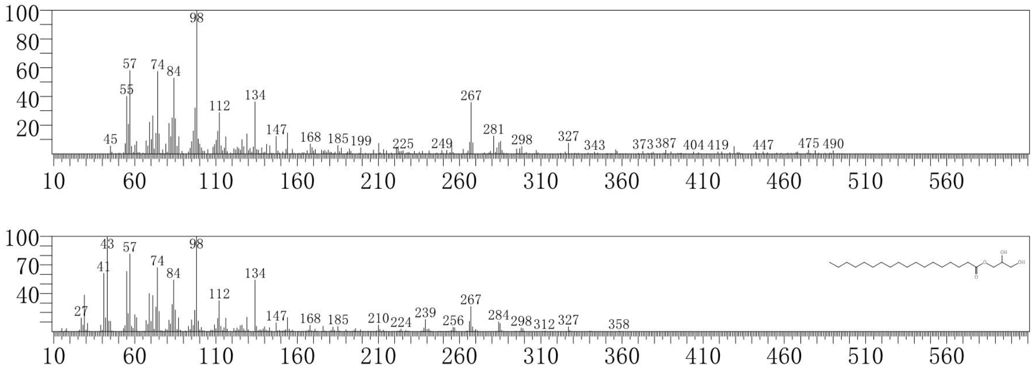

Figure S23. GC-MS spectrum of component **22**

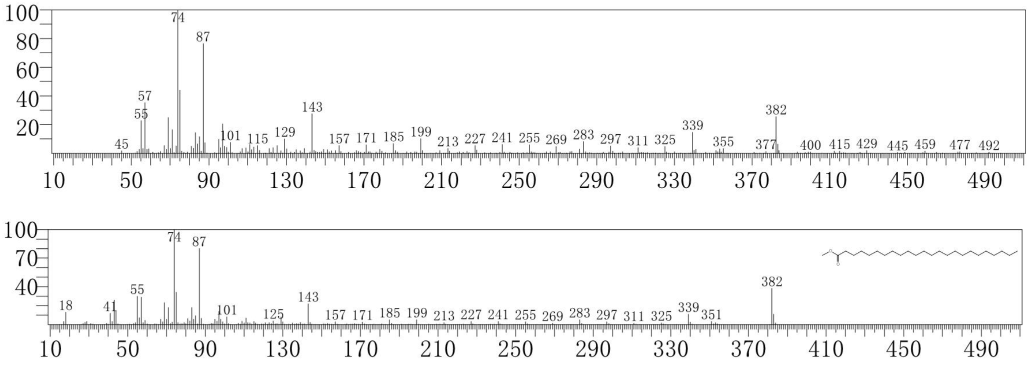

Figure S24. GC-MS spectrum of component **23**

Table S1 Molecular interactions of the α-glucosidase (3A4A) active site with compounds **1** and **2**.

Compounds	Binding energy (kcal/mol)	H-bond	Hydrophobic interaction			Others
			Pi-Alkyl	Pi-Pi T-shaped	Pi-Pi Stacked	Pi-Anion
**1**	-2.41	LYS466, THR83, TRP81, SER65, PRO66, GLN67, ARG413, LYS406		TRP36	TYR470	
**2**	-3.55	SER304, GLY309, PRO312, THR310, SER240, HIS280	PRO312, HIS280, ARG315	TYR158, HIS280		ASP242
Acarbose	-4.53	SER304, ASN302, GLU332, SER241, LYS156, ARG315, PRO312				
